# Mean platelet volume in children with hepatitis A

**DOI:** 10.1186/s41043-016-0070-0

**Published:** 2016-10-06

**Authors:** Fatih Akın, Ahmet Sert, Şükrü Arslan

**Affiliations:** 1 Department of Pediatrics, Necmettin Erbakan University, Meram Medical Faculty, 42080 Konya, Turkey; 2 Department of Pediatric Cardiology, Konya Training and Research Hospital, Konya, Turkey; 3 Department of Pediatric Nephrology, Konya Training and Research Hospital, Konya, Turkey

**Keywords:** Viral infection, Child, Inflammation, Cytokines

## Abstract

**Background:**

Mean platelet volume (MPV), which is commonly used as a measure of platelet size, indicates the rate of platelet production and platelet activation. We aimed to evaluate the mean platelet volume in children with hepatitis A.

**Methods:**

In this retrospective case-controlled study, the study population consisted of 62 children with hepatitis A and 62 healthy control subjects.

**Results:**

MPV values, aspartate transaminase (AST), and alanine transaminase (ALT) levels on admission were significantly increased in patients with hepatitis A when compared to controls whereas white blood cell (WBC) counts were significantly lower. Two weeks after admission, the MPV values showed a significant decrease from 9.47 ± 1.62 to 8.84 ± 1.48 fL in patients with hepatitis A, but these values were still significantly higher than the controls. There was a significant difference in terms of MPV, WBC, AST, and ALT values between the controls and the patient group 2 weeks after admission.

**Conclusions:**

This study is the first to evaluate the MPV levels in children with hepatitis A. MPV values were found to be increased in children hospitalized with hepatitis A.

## Background

Hepatitis A is inflammation of liver that results from infection with the hepatitis A virus (HAV). It can range in severity from a mild illness lasting a few weeks to a severe illness lasting several months. During hepatic viral infection, various cytokines play roles in viral clearance and tissue damage. Changes in cytokine activities might favor viral persistence and consequently disease progression [1].

Mean platelet volume (MPV), which is commonly used as a measure of platelet size, indicates the rate of platelet production and platelet activation [2]. It also reflects the existence of thrombotic and inflammatory processes. Previously, MPV values have been studied in hepatic diseases such as non-alcoholic fatty liver disease (NAFLD), hepatitis B and hepatitis C [3–8]. Increased levels of MPV in chronic hepatitis B and C were associated with the degree of the liver inflammation and liver fibrosis [6–8]. Higher MPV in NAFLD patients was reported to indicate possible cardiovascular disease risk increase [3].

Different serum cytokine profiles in distinct clinical profiles of hepatitis A have been described [1]. MPV levels may reflect the cytokine levels and the inflammatory process in hepatitis A. To the best of our knowledge, MPV values have not been previously assessed in patients with hepatitis A. We aimed to investigate the MPV values in patients with hepatitis A at an early stage of illness and recovery phase and compared results with healthy control subjects.

## Methods

### Study population

Data of all children who were hospitalized with diagnosis of hepatitis A in Konya Training and Research Hospital Pediatrics Clinic between January 2012 and January 2013 were retrospectively reviewed. These patients had required hospitalization because of severe nausea and vomiting. The patients were considered eligible for participation if the clinical case definition of hepatitis A of the Centers for Disease Control and Prevention, which is “an acute illness with discrete onset of symptoms and either jaundice or elevated serum aminotransferase levels and a positive immunoglobulin M antibody to HAV (anti-HAV) serologic result”, was met [9]. Patients with any other infectious disease or chronic disease including hematologic and hepatic diseases were not enrolled into the study.

The following data were collected by using the computerized patient database: complete blood count including white blood cell (WBC), platelet counts, activated partial thromboplastin time (aPTT), prothrombin time (PT), MPV, aspartate transaminase (AST), alanine transaminase (ALT), and bilirubin, at the time of diagnosis and 2 weeks after admission.

A total of 62 children with hepatitis A and 62 age- and gender-matched healthy controls were enrolled in the study. The healthy subjects were children who applied to hospital for routine check-up. Control group subjects were recruited from hospital records of these children. Children with any sign of infection or systemic illness were excluded from the control group. The laboratory parameters of the healthy children were recorded from the same computerized database. The complete blood count analyses were performed in the same Coulter analyzer (Sysmex XE-2100, Sysmex Corporation, Kobe, Japan) in the central laboratory of our institution. Standard tubes with constant amount of ethylenediaminetetraacetic acid were used. Standard liver function tests (ALT, AST) and bilirubin levels were measured with an auto analyzer. Anti-HAV was analyzed by a chemiluminescent microparticle immunoassay method.

### Statistical analysis

Data were reported as mean ± standard deviation. If not normally distributed, parameters were presented as median (minimum-maximum). The Kolmogorov-Smirnov test was applied to check the distribution of parameters. Differences in the means of variables were evaluated using both parametric and nonparametric tests depending on the distribution of the variables. Paired samples *t* test or Mann-Whitney *U* test was used to compare groups. Paired samples *t* test or Wilcoxon signed-rank test was used to compare values of the patients with hepatitis A, on admission and 2 weeks after admission. Results were considered significant if *p* < 0.05. Statistical analysis was performed using a computer software package (SPSS for Windows, version 15.0).

## Results

Table 1 shows clinical characteristics of the patients on admission and 2 weeks after the admission and characteristics of the controls. MPV values and AST and ALT levels on admission were significantly increased in patients with hepatitis A when compared to the controls (*p <* 0.0001, *p <* 0.0001, and *p <* 0.0001, respectively) whereas WBC counts were significantly lower (*p =* 0.023). Two weeks after admission, we found a significant decrease in the MPV values, from 9.47 ± 1.62 to 8.84 ± 1.48 fL, in patients with hepatitis A, but these values were still significantly higher than the controls. There was a significant difference in terms of MPV, WBC, AST, and ALT values between the controls and the patient group 2 weeks after admission (*p <* 0.0001, *p =* 0.021, *p* = 0.004, and *p* < 0.0001, respectively).

**Table 1:** Demographic and laboratory characteristics of patients with hepatitis A on admission (A), 2 weeks after admission (B), and controls

				p value		
Characteristics	Healthy controls	A	B	Control vs. A	Control vs. B	A vs. B
Number of patients	62	62	62			
Age, years	9.1 ± 4.3	9.6 ± 3.6	9.6 ± 3.6			
Male/female ratio	30/32	30/32	30/32			
WBC (count/mm^3^)^a^	7800 (4800–11,000)	5440 (2100–27,400)	7197 (3900–6200)	*0.023*	*0.021*	NS
Platelet counts (count × 10^3^/mm^3^)^a^	336 (176–967)	278 (22–748)	298 (69–554)	NS	NS	0.042
MPV (fL)	8.28 ± 1.13	9.47 ± 1.62	8.84 ± 1.48	*<0.0001*	*<0.0001*	*0.005*
AST (IU/L)^a^	27(11–33)	927 (34–4145)	33 (16–519)	*<0.0001*	*0.004*	*<0.0001*
ALT (IU/L)^a^	14 (6–36)	1250 (36–4113)	35 (6–1358)	*<0.0001*	*<0.0001*	*<0.0001*
T. bil (IU/L)^a^	–	1 (0.3–17.2)				
D. bil(IU/L)^a^	–	0.67 (0.1–7.7)				
PTT	–	29.1 (18.1–46.9)				
PT	–	13.7 (10.7–35.1)				

Parametric values were expressed as means with standard deviation. Significance was determined by *p* < 0.05

*AST* aspartate transaminase, *ALT* alanine transaminase, *PT* prothrombin time, *aPTT* activated partial thromboplastin time, *MPV* mean platelet volume, *WBC* white blood cell, *T.bil* total bilirubin, *D.bil* direct bilirubin, *NS* not significant

^a^If not normally distributed, values were presented as median (minimum-maximum)

## Discussion

To the best of our knowledge, this is the first study to evaluate the MPV values in children with hepatitis A. We demonstrated that MPV values were higher in children with hepatitis A compared to healthy control subjects.

MPV is one of the platelet function marker that reflects platelet production rate and stimulation. It also reflects the existence of thrombotic and inflammatory processes. MPV elevates when both platelet production and destruction are increased, which is mediated by cytokines such as interleukin (IL)-3, IL-6, and thrombopoietin. IL-3 or IL-6 regulate megakaryocyte ploidy and can contribute the production of more reactive and larger platelets [2]. Gasparyan et al. reported that an increased MPV is a reflection of both proinflammatory and prothrombotic conditions, where thrombopoietin and numerous inflammatory cytokines (e.g., IL-1, IL-6, tumor necrosis factor alpha (TNF-α)) regulate thrombopoiesis. They also mentioned in their review that an increase in the entry of young platelets into circulation from the bone marrow causes an increase in MPV values, due to increased inflammatory cytokines in patients with inflammatory diseases such as familial Mediterranean fever and Behçet disease. They suggested that MPV could be a useful biomarker for inflammation and thrombosis and increases of MPV values are associated with low-grade inflammation [10]. In our study 2 weeks after admission, we found a significant decrease in the MPV values, from 9.47 ± 1.62 to 8.84 ± 1.48 fL, in patients with hepatitis A, but these values were still significantly higher than the controls. As the clinical course of hepatitis A takes several weeks, this finding in our study may reflect the inflammatory process in hepatitis A.

Different serum cytokine profiles in distinct clinical profiles of hepatitis A have been described [1]. In contrast with most infectious diseases, acute viral hepatitis is characterized by a lack or only a moderate degree of acute phase reaction, which takes an asymptomatic course, especially in children [11]. Torre et al. reported that serum levels of IL-1a, IL-6, and TNF-α were increased during the acute phase of the disease. This elevation was explained by the fact that the liver is an important site of production as well as a major site of clearance of these cytokines [12]. Müller et al. demonstrated that production of TNF by peripheral blood monocytes was reduced in patients with acute hepatitis B infection but not in those with acute hepatitis A [13]. On the other hand, Muto et al. reported normal levels of TNF-α in patients with acute hepatitis A or B without fulminant liver failure [14]. Müller et al. studied IL-6 and IL-1 levels in patients with hepatitis A in two different studies. He demonstrated normal levels of IL-6 and severely decreased during the first week and gradually increased IL-1 levels during the further course of hepatitis A [11, 15]. Fierro et al. reported an over expression of TNF-α, IL-1, and IL-6 in children with HAV-induced intermediate liver damage while minor liver damage was characterized by increase of IL-8 and transforming growth factor beta, suggesting that an imbalance in the inflammatory cytokines occurs during the course of hepatitis A [1]. In the study of Szkaradkiewicz et al., the course of acute self limited hepatitis B was associated with preferential T helper1-type response, which was manifested by elevated production of interferon gamma and IL-2 [16]. We think that increased levels of proinflammatory cytokines had caused elevation of MPV in patients with hepatitis A in the current study.

Almost all studies regarding MPV values in hepatic diseases demonstrate increased MPV values. An increase in MPV has been reported in patients infected with hepatitis B virus and hepatitis C virus and was related to the increase in the entry of newly produced platelets into circulation, which are larger in volume. The breakdown of platelets in the spleen and increased levels of IL-6 production secondary to inflammation in the fibrosis process were thought to contribute increased MPV in patients with chronic hepatitis B (CHB) [6–8]. Ceylan et al. also observed an increase in MPV in patients with chronic hepatitis B predicting the degree of liver inflammation [6]. An increase in MPV is also reported in chronic hepatitis B patients with inactive disease [5]. Ekiz et al. reported a correlation of MPV and liver fibrosis and hypothesized that increased levels of IL-6 production secondary to inflammation in the fibrosis process may cause increased MPV in CHB [8]. A study conducted on patients with chronic hepatitis C (CHC) and CHC with advanced fibrosis reported an increase of MPV at the time of diagnosis and in the post-treatment period. This was attributed to be an early sign of advanced liver fibrosis that is warning sign for forthcoming decompensation in these patients [7]. The results of Ozhan et al.’s study showed also that MPV was increased in patients with NAFLD, which is serving as a surrogate marker of cardiovascular risk in this clinical setting [3]. Cho et al. report that thrombopoietin, which is synthesized in the liver could play a role to increase MPV in hepatic diseases. Moreover, several cytokines, inflammatory mediators, and growth factors may affect MPV [4]. In addition to elevation of cytokine levels, thrombopoietin might also have contributed the increase of MPV in our patients with hepatitis A.

We demonstrated increased MPV values in patients hospitalized with hepatitis A for the first time in literature. In view of the results of the studies discussed above, we speculate that increased proinflammatory cytokines and thrombopoietin may have contributed to elevation of MPV in patients with hepatitis A. The decrease of MPV after 2 weeks in the recovery phase also supports this speculation.

### Limitations

Our study had some limitations, such as its retrospective design and lack of data of the cytokine levels of the study group. Additionally, this study consists children with hepatitis A who were hospitalized; thus, further studies are needed for outpatients and the adult age group.

## Conclusions

Our study demonstrated that MPV values are increased in children hospitalized with hepatitis A. However, prospective studies with larger number of patients are needed to assess the mechanism of increased MPV values in hepatitis A.

## Abbreviations

ALT: Alanine transaminase
Anti-HAV: Antibody to HAV
aPTT: Activated partial thromboplastin time
AST: Aspartate transaminase
CHB: Chronic hepatitis B
CHC: Chronic hepatitis C
HAV: Hepatitis A virus
IL: Interleukin
MPV: Mean platelet volume
NAFLD: Non-alcoholic fatty liver disease
PT: Prothrombin time
TNF-α: Tumor necrosis factor alpha
WBC: White blood cell

## References

[1] Fierro NA, Escobedo-Melendez G, De Paz L, Realpe M, Roman S, Panduro A (2012). Cytokine expression profiles associated with distinct clinical courses in hepatitis A virus-infected children. Pediatr Infect Dis J.

[2] Colkesen Y, Muderrisoglu H (2012). The role of mean platelet volume in predicting thrombotic events. Clin Chem Lab Med.

[3] Ozhan H, Aydin M, Yazici M, Yazgan O, Basar C, Gungor A (2010). Mean platelet volume in patients with non-alcoholic fatty liver disease. Platelets.

[4] Cho SY, Lee A, Lee HJ, Suh JT, Park TS (2012). Mean platelet volume in Korean patients with hepatic diseases. Platelets.

[5] Turhan O, Coban E, Inan D, Yalcin AN (2010). Increased mean platelet volume in chronic hepatitis B patients with inactive disease. Med Sci Monit.

[6] Ceylan B, Fincanci M, Yardimci C, Eren G, Tözalgan Ü, Müderrisoğlu C (2013). Can mean platelet volume determine the severity of liver fibrosis or inflammation in patients with chronic hepatitis B?. Eur J Gastroenterol Hepatol.

[7] Purnak T, Olmez S, Torun S, Efe C, Sayilir A, Ozaslan E (2013). Mean platelet volume is increased in chronic hepatitis C patients with advanced fibrosis. Clin Res Hepatol Gastroenterol.

[8] Ekiz F, Yüksel O, Koçak E, Yılmaz B, Altınbaş A, Çoban S (2011). Mean platelet volume as a fibrosis marker in patients with chronic hepatitis B. J Clin Lab Anal.

[9] National Notifiable Diseases Surveillance System. Centers for Disease Control and Prevention (US). Case definition of hepatitis A. 2012. https://wwwn.cdc.gov/nndss/conditions/hepatitis-a-acute/casedefinition/2012/. Accessed 9 Sept 2015.

[10] Gasparyan AY, Ayvazyan L, Mikhailidis DP, Kitas GD (2011). Mean platelet volume: a link between thrombosis and inflammation?. Curr Pharm Des.

[11] Müller C, Gödl I, Ahmad R, Wolf HM, Mannhalter JW, Eibl MM (1989). Interleukin-1 production in acute viral hepatitis. Arch Dis Child.

[12] Torre D, Zeroli C, Giola M, Ferrario G, Fiori GP, Bonetta G (1994). Serum levels of interleukin-1 alpha, interleukin-1 beta, interleukin-6, and tumor necrosis factor in patients with acute viral hepatitis. Clin Infect Dis.

[13] Müller C, Zielinski CC (1990). Impaired lipopolysaccharide-inducible tumor necrosis factor production in vitro by peripheral blood monocytes of patients with viral hepatitis. Hepatology.

[14] Muto Y, Nouri-Aria KT, Meager A, Alexander GJ, Eddleston AL, Williams R (1988). Enhanced tumour necrosis factor and interleukin-1 in fulminant hepatic failure. Lancet.

[15] Müller C, Zielinski CC (1992). Interleukin-6 production by peripheral blood monocytes in patients with chronic liver disease and acute viral hepatitis. J Hepatol.

[16] Szkaradkiewicz A, Jopek A, Wysocki J, Grzymislawski M, Malecka I, Woźniak A (2003). HBcAg-specific cytokine production by CD4 T lymphocytes of children with acute and chronic hepatitis B. Virus Res.

